# Responsivity of Fractal Nanoparticle Assemblies to Multiple Stimuli: Structural Insights on the Modulation of the Optical Properties

**DOI:** 10.3390/nano12091529

**Published:** 2022-05-01

**Authors:** Angela Capocefalo, Thomas Bizien, Simona Sennato, Neda Ghofraniha, Federico Bordi, Francesco Brasili

**Affiliations:** 1 Institute for Complex Systems (ISC-CNR), National Research Council, 00185 Rome, Italy; simona.sennato@roma1.infn.it (S.S.); neda.ghofraniha@roma1.infn.it (N.G.); federico.bordi@roma1.infn.it (F.B.); 2 Department of Physics, Sapienza University of Rome, 00185 Rome, Italy; 3 Synchrotron SOLEIL, L’Orme des Merisiers, Saint-Aubin, BP 48, CEDEX, 91192 Gif-sur-Yvette, France; thomas.bizien@synchrotron-soleil.fr

**Keywords:** gold nanoparticles, proteins, patchy colloids, self-assembly, nanosensor, hybrid nanomaterials, biosensing, Small Angle X-ray Scattering (SAXS), plasmonic resonance

## Abstract

Multi-responsive nanomaterials based on the self-limited assembly of plasmonic nanoparticles are of great interest due to their widespread employment in sensing applications. We present a thorough investigation of a hybrid nanomaterial based on the protein-mediated aggregation of gold nanoparticles at varying protein concentration, pH and temperature. By combining Small Angle X-ray Scattering with extinction spectroscopy, we are able to frame the morphological features of the formed fractal aggregates in a theoretical model based on patchy interactions. Based on this, we established the main factors that determine the assembly process and their strong correlation with the optical properties of the assemblies. Moreover, the calibration curves that we obtained for each parameter investigated based on the extinction spectra point out to the notable flexibility of this nanomaterial, enabling the selection of different working ranges with high sensitivity. Our study opens for the rational tuning of the morphology and the optical properties of plasmonic assemblies to design colorimetric sensors with improved performances.

## 1. Introduction

The controlled assembly of gold nanoparticles (AuNPs) has proved to be a promising route for the fabrication of novel bottom-up plasmonic nanomaterials with selectable features to be employed in diverse applications, ranging from electronics to medicine [1]. Particularly attractive are the optical properties of these systems, that can be finely tuned in a broad spectral range by acting on the clustering process. These properties stem from the excitation of localized surface plasmons, that results in the confinement of enhanced electromagnetic fields at the particle surface and leads to strong extinction at the resonant wavelength (Localised Surface Plasmon Resonance, LSPR) [2]. The LSPR of AuNPs-based systems can be tailored by the morphology of the assemblies, by the number of constituent AuNPs and by the distribution of the interparticle distances within the aggregates [3,4]. In fact, from dimers to more complex nanoarchitectures, when AuNPs are in close proximity, the single localized plasmonic modes hybridize into new coupling bands shifted at higher wavelengths, visually resulting in a color change of the colloidal dispersion [5]. The spectral weights of these coupled modes is determined by the structure and size of the clusters. Owing to these flexible optical properties, AuNPs assemblies are increasingly employed as versatile platforms for advanced applications in optical and chemical sensing [6], especially for colorimetric assays [7] and spectroscopic detection [8,9]. On top of that, a further peculiarity of gold is its chemical stability, that results in low toxicity of AuNPs thus enabling their application in biosensing [10] and nanomedicine [11,12]. In this context, the toxic side effects of AuNPs related to concentration, size and molecular capping have been extensively evaluated [13]. It has been reported that the toxicity of citrate-capped AuNPs is strongly reduced if their diameter exceeds 40 nm [14,15]; therefore, in the present study, we focused on citrate-capped AuNPs with diameter of 60 nm, that show a high biocompatibility [16].

The main strategy for tuning the optical properties of plasmonic aggregates through the clustering process is to decorate the surface of the AuNPs with stimuli-responsive moieties [17]. In this way, the self-assembly can be controlled relying on changes of the interaction potential between the colloidal particles induced by environmental parameters such as pH, ionic strength and temperature.

Among the available stimuli-responsive compounds, proteins and DNA, that are characterized by programmable inter-molecular interactions and responsiveness to external stimuli, allow for realizing self-assembled hybrid systems with the desired features [18,19,20]. In addition, biological macromolecules have the huge advantage of being intrinsically biocompatible and biodegradable, allowing their use even for in vivo biosensing [21,22,23]. Protein- and DNA- mediated aggregation of AuNPs has been successfully employed in the realization of colorimetric assays for the recognition of biomolecular processes such as DNA hybridisation [24] and detection of target molecules such as exosomal proteins [4] and heparin [25].

To take advantage of this potential, a detailed analysis aimed at establishing the exact correspondence between the optical response and the structural modification of the plasmonic aggregates is mandatory. Here, we propose an in-depth investigation of the structural and optical properties of a self-assembled hybrid plasmonic material by combining Small Angle X-ray Scattering (SAXS) and extinction spectroscopy. We adopt an electrostatic approach for the realization of protein-decorated colloidal particles by employing anionic AuNPs. In a previous study, we demonstrated that the resulting nanomaterial shows effective antibacterial activity [19], however, as a further advantage, the responsivity to external stimuli provided by the protein makes this system promising to be employed as nanosensor. In this work, we provide a detailed analysis of the protein-mediated aggregation process by relating the morphology of the complexes—in terms of their fractal dimension and interparticle distance—with their optical response at varying protein concentration, pH and temperature. Based on extinction spectra, we build a calibration curve for each one of these parameters, in order to gain the full control on these external stimuli which is pivotal for the realization of effective sensors.

## 2. Materials and Methods

### 2.1. Materials

Citrate-stabilised AuNPs with nominal diameter of 60 nm and number density of $2.6\times 10^{10}$ mL${}^{-1}$ were provided by Ted Pella (Redding, CA, USA). Chicken egg white lysozyme powder (MW 14.4 kDa, purity > 90%) was provided by Sigma-Aldrich. Sodium citrate buffer at pH 6.5 was provided by Merck Millipore (Burlington, MA, USA). All the chemicals employed in sample preparation were purchased from Sigma-Aldrich (St. Louis, MO, USA) and used without further purification.

### 2.2. Sample Preparation

A stock solution was prepared by dissolving the lysozyme powder in 20 mM sodium citrate buffer to keep the pH at 6.5. Protein solutions at different concentrations were prepared by diluting the initial protein solution in a proper volume of the same buffer. Lysozyme-AuNPs samples at different protein-AuNP number ratios ξ were therefore obtained by adding to each protein solution an equal volume of the AuNPs stock solution. In the analysed samples lysozyme concentration ranges between 17 nM and 215 nM, to obtain ξ values between 800 and 10,000. After mixing the two components, we set an incubation time to left AuNPs clusters grow up to their equilibrium size. The analysis aimed at identify the growth times of samples prepared at different ξ are reported in Section S1 of the Supplementary Materials.

Measurements as a function of pH were performed by adjusting the pH of the solutions of lysozyme-AuNPs samples by adding aliquots of hydrochloric acid (HCl) or sodium hydroxide (NaOH) for obtaining solutions at acidic and basic pH, respectively. An equilibration time of at least 5 min was scheduled before measuring the samples.

### 2.3. Dynamic Light Scattering

The size of AuNPs clusters was characterized in terms of their hydrodynamic diameter $2R_{H}$ by Dynamic Light Scattering (DLS) measurements. Experiments were performed using a NanoZetaSizer apparatus (Malvern Instruments LTD, Worcester, UK), equipped with a 5 mW He-Ne laser emitting at 633 nm. The scattered light was collected at an angle of 173°. Decay times were extrapolated from the acquired intensity autocorrelation functions using the CONTIN algorithm. Decay times were used to determine the distribution of the diffusion coefficients *D*, which are converted in the intensity-weighted distributions of the hydrodynamic diameter using the Stokes–Einstein relationship $R_{H}=k_{B}T/6\pi \eta D$, where $k_{B}T$ is the thermal energy and η the water viscosity. The collected data were analyzed using the Zetasizer software provided with the instrument.

The reported results are obtained on measurements performed after the incubation time, of at least 5 min, scheduled for samples preparation or pH adjustment. The minimum time needed to reach the equilibrium size of clusters, which depends on the lysozyme-AuNPs molar ratio, was evaluated as described in Section S1 of Supplementary Materials. For experiments at different temperatures, after every temperature change the samples were kept thermalizing for 5 min before acquisition. In any case, we considered samples as stabilized when we could perform at least three consecutive measurements that yielded comparable hydrodynamic diameter distributions. The reported values and the associated errors are obtained by averaging the center values of these distributions and by the corresponding standard deviation.

### 2.4. Extinction Spectroscopy

Extinction spectra were acquired in the range of wavelengths from 200 to 1100 nm using a double ray spectrophotometer V-570 (Jasco, Tokyo, Japan), equipped with a Peltier thermostatted holder EHC-505 (Jasco). The spectral resolution of the instrument is of 0.1 nm in the UV–Visible and 0.5 nm in the near-infrared. Experiments at different protein concentrations and pH were performed at 25 °C, after an equilibration time, of at least 5 min and evaluated for each lysozyme-AuNPs molar ratio, from samples preparation or pH adjustment. For experiments at different temperatures, after every temperature change the samples were kept thermalizing for 5 min before acquisition. In any case, we considered samples as stabilized when we could perform at least three consecutive measurements that yielded the same spectrum. The spectra here reported are normalized to the extinction at 400 nm, where the absorption coefficient is proportional to the molar concentration of Au(0) in the sample [26].

### 2.5. Scanning Electron Microscopy

SEM images were acquired using an Auriga 405 microscope (Zeiss, Jena, Germany), with an extracting voltage of 15 kV and 350× magnification. For sample deposition, silicon substrates were functionalized by incubating a 3% ethanol solution of (3-Aminopropyl)triethoxysilane (APTES) for 3 h. Subsequently, 50 µL of the sample solution was incubated for 5 min on the substrate and then removed by gently rinsing with MilliQ water. The samples were finally dried under gentle nitrogen flow.

### 2.6. Small Angle X-ray Scattering

The structure of the AuNPs clusters in solution was investigated by Small Angle X-ray Scattering (SAXS). Experiments were performed at Synchrotron SOLEIL (Saint Aubin, France), SWING beamline. For measurements, samples were filled in capillaries (1.5 mm diameter) and placed at the sample-to-detector distance of 6.5 m. The exposure time for acquisitions was set to 1 s and 30 scattering patterns were acquired for each sample. Scattering patterns were recorded using a two-dimensional EigerX 4-M detector (Dectris, Baden, Switzerland) at 12 keV, allowing measurements in the range of *q*-vector—defined as q=(4π/λ)sinθ, where 2θ is the scattering angle—from 0.001 to 0.18 Å${}^{-1}$. For intensity background subtraction, scattering patterns of an empty capillary and of a capillary filled with Milli-Q water were recorded. Experiments at different protein concentrations and pH were performed at 25 °C, after an equilibration time of about 20 min from samples preparation or pH adjustment. For measurements as a function of temperature, a Huber Ministat 125 thermostat was employed and experiments were conducted at selected temperatures from 25 °C to 80 °C. For each temperature, a thermalization time of 5 min was scheduled before acquisition. The processing and averaging of the scattering patterns were performed by the software Foxtrot (SOLEIL software group and SWING beamline). When averaging, any scattering curve not perfectly superimposed with the overall set acquired, due to possible residual equilibration or other experimental perturbations, was discarded.

For a collection of particles, the scattered intensity I(q) can be expressed in terms of the form factor P(q) of single particles and of the structure factor S(q) of the system:

(1) $$I\left(q\right)=nv^{2}\Delta \rho^{2}P\left(q\right)S\left(q\right),$$

where *n* and *v* are the number density and the volume of the scattering particles, and Δρ is the contrast in electron density between particles and solvent. Here, P(q) describes the ensemble averaged shape of scattering objects in solution, whereas S(q) accounts for the interference introduced by interparticle correlations. For a dilute system of non-interacting scatterers, S(q)=1 [27]. In our experiments, the form factor is directly measured on the stock solution of AuNPs. To derive the radius $R_{0}$ of AuNPs, the obtained curve was fitted to a spherical form factor model [27], assuming a log-normal particle size distribution. To derive the structure factor of AuNPs clusters, we divided the scattered intensity measured on each sample by the form factor P(q). The obtained S(q) curves were fitted to a sticky hard spheres model [28], defined by a Percus–Yevick approximation with an attractive square well potential [29,30], to extrapolate the effective radius *R* of the interacting colloids. Data analysis was performed by the software SasView, version 5.04 [31].

## 3. Results

For clarity of presentation, in this section we report the investigation of the morphological and optical properties of AuNPs clusters obtained by protein-mediated aggregation separately for each parameter investigated: protein concentration, temperature and pH of the solution.

### 3.1. Aggregates at Different Protein Concentrations

**Figure 1:**
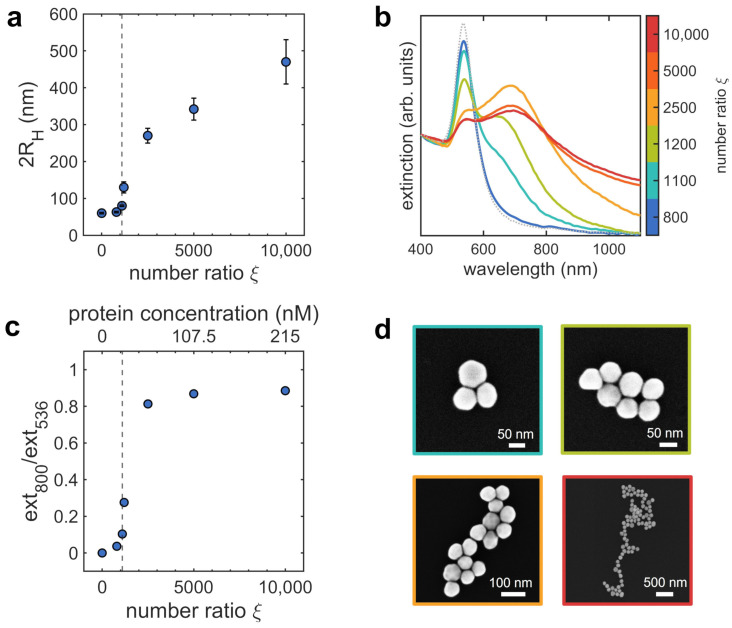
Characterization of AuNPs clusters obtained for different protein concentrations (the lysozyme-AuNPs number ratio ξ is represented by the colorbar), performed at 25 °C and pH 6.5: morphology and optical properties. (**a**) hydrodynamic diameter $2R_{H}$, values and errors are the mean and the standard deviation of at least 3 measurements; (**b**) extinction spectra, the spectrum of bare AuNPs is shown by dashed gray line for comparison; (**c**) calibration curve obtained for protein concentration: the experimental parameter employed for quantifying the aggregation of AuNPs, namely the ratio between the extinction values measured at 800 and 536 nm, is reported as a function of protein concentration. The vertical dashed lines in panels a and c indicate the onset of the aggregation. (**d**) Representative SEM images of clusters for ξ=1100, ξ=1200, ξ=2500 and ξ = 10,000.

We studied the protein-mediated aggregation of AuNPs at varying the number ratio ξ between the two species in the colloidal dispersion at 25 °C and pH 6.5. To this aim, we kept the AuNPs number density fixed at $1.3\times 10^{10}$ mL${}^{-1}$ and varied the lysozyme concentration between 17 and 215 nM, corresponding to ξ values ranging from 800 to 10,000. The characterization of the formed clusters by DLS, extinction spectroscopy and SEM is reported in Figure 1.

DLS experiments reported in Figure 1a show the self-limited aggregation of AuNPs into assemblies whose average size varies in dependence on ξ. We also analyzed the temporal evolution of the size distributions over a period of 100 min (Section S1 of Supplementary Materials), assessing the self-limited nature of the aggregation process. At low number ratios (ξ<1100), the value of the hydrodynamic diameter is almost constant around 60 nm, thus indicating that the sparsely protein-decorated AuNPs do not aggregate. Upon increasing the number ratio, as the adsorption of the protein to AuNPs increases, the steep variation in the hydrodynamic diameter trend for ξ≥1100 highlights the formation of AuNPs aggregates, that reach a maximum size of ∼500 nm for the highest number ratio investigated. Extinction spectra of the complexes at varying ξ are reported in Figure 1b. The optical properties showed excellent stability for 1 h after sample preparation, coherently with DLS measurements; afterwards weak sedimentation effects are observed for ξ≥2500 (Section S1 of Supplementary Materials). At the lowest number ratio (ξ=800), the shape of the extinction spectrum of the sample is comparable to that of the AuNPs stock solution, confirming that the aggregates are not yet formed in solution. Starting from ξ=1100, a shoulder at higher wavelengths appears in the extinction spectra, accompanied by a progressive broadening and shift of the LSPR. These spectral modifications are indicative of plasmon coupling. In fact, when AuNPs are in close proximity (few nanometers), their extinction spectrum acquires a new band, red-shifted significantly from that of isolated nanoparticles, whose intensity increases with the number of interacting AuNPs [5]. The position and width of this band depend on the average interparticle distance and on its statisical distribution within clusters [32]. Such spectral modifications are more pronounced with increasing number ratio, with the extinction spectra widened in the near infrared region. This is consistent with the clusters growth highlighted by DLS. At the highest number ratios, extinction spectra do not evolve anymore, as expected for large clusters, in which the extinction is dominated by the contribution of long chains of nanoparticles [33,34].

DLS and extinction spectroscopy experiments evidence the close correspondence between the progressive aggregation of the colloids and their optical properties [19]. To have an experimental parameter that allows us to monitor the aggregation process and the subsequent variations of the morphology of the clusters through the spectral changes, we define the ratio between the extinction intensities at 800 and 536 nm, that weights the contributions to the spectra of interacting and non-interacting plasmon modes. The intensity at 800 nm, that increases when AuNPs assemble and the coupling of plasmonic modes becomes more relevant, is the marker parameter for AuNPs aggregation, while the intensity at 536 nm corresponds to the maximum of the extinction peak of single AuNPs (see the spectrum reported in gray in Figure 1b). The analysis of the time evolution of this parameter is reported in Section S1 of the Supplementary Materials, revealing excellent stability even in the case of the larger clusters when a hint of sedimentation is observed. The obtained curve as a function of the protein concentration is reported in Figure 1c, highlighting the onset of the aggregation at ξ=1100, corresponding to a protein concentration of 24 nM.

Representative SEM images of the samples are reported in Figure 1d. We accurately chose and optimized the procedure used for depositing samples onto silicon substrates reported in Section 2.5 in order to minimize possible alterations of the clusters structure during the adhesion to the silicon surface and to avoid undesired aggregation [35]. SEM micrographs show the formation of assemblies where the average number of AuNPs per cluster increases with ξ. Small clusters (ξ=1100 and ξ=1200) appear compact, while larger ones are less regular and some protruding branches appear.

**Figure 2:**
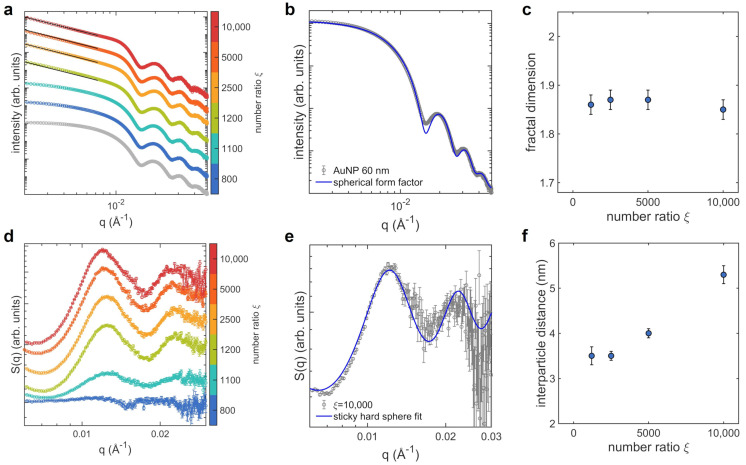
SAXS analysis of the AuNPs clusters morphology as a function of the protein concentration (the lysozyme-AuNPs number ratio ξ is represented by the colorbar), performed at 25 °C and pH 6.5: (**a**) SAXS curves (circles, the curve in gray is acquired on the AuNPs stock solution) and power-law decay fitting curves (black lines, only for curves at ξ≥1200 that show a clear power-low decay in the *q*-range from 0.002 to 0.0072 Å${}^{-1}$); (**b**) fit of the SAXS curve of the AuNPs stock solution (gray circles) to a spherical form factor (blue line); (**c**) fractal dimension as a function of ξ; (**d**) structure factors at varying ξ; (**e**) representative fit of a structure factor S(q) (gray circles, sample at ξ = 10,000) to a sticky hard spheres model (blue line); (**f**) interparticle distance as a function of ξ. SAXS curves and structure factors are shifted vertically for clarity.

Moving from the results provided by DLS and SEM and with the aim of establishing a connection between the structure of AuNPs clusters and the observed modulation of the optical properties, we performed a detailed SAXS analysis of the clusters in solution, as reported in Figure 2. We acquired SAXS curves on samples prepared at the same number ratios considered for extinction spectroscopy (Figure 2a). The shape of the scattered intensity curves in the low-*q* region ($q<5\times 10^{-3}$Å${}^{-1}$) allows us to easily recognize AuNPs aggregation. In fact, the scattering curves of non-aggregated AuNPs show a clear plateau in this region and the overall shape overlaps quite well the typical scattered intensity of non-interacting spheres (Figure 2b). The presence of larger objects in solution, namely AuNPs aggregates, is identified by a steeper negative slope in the same low-*q* region. In our case, for ξ=800 the amount of protein that adsorbs to AuNPs is not sufficient to perturb the stability of the colloidal dispersion. For ξ=1100, the plateau is no more clearly reached and, starting from ξ=1200, AuNPs aggregation is clearly observed, consistently with DLS and with the appearance of coupled plasmon modes in the extinction spectra.

We analyzed SAXS curves focusing on the mass fractal dimension $D_{f}$ of clusters and on the interparticle distance *d*. The first parameter is commonly used to describe the geometry of complex and disordered structures, characterized by recursive patterns at different length scales [36,37]. It measures the scaling of the mass *M* with the size of the aggregate and is defined by the expression $M\propto R^{D_{f}}$, where *R* is any linear measure of the size [36]. In this respect, the fractal dimension is strictly linked to the global topology of plasmonic nanoparticles’ aggregates and therefore it determines their optical properties [38,39,40].

The linear decrease of I(q) in the log-log plot, observed for ξ≥1200 in the *q*-range from $3\times 10^{-3}$ to $8\times 10^{-3}$ Å${}^{-1}$, is typical of mass fractal objects [36]. We therefore fitted the scattered intensity to a power law decay $I\left(q\right)\propto q^{-D_{f}}$ in the selected *q*-range [41] to derive the fractal dimension. The obtained fractal dimension values, plotted as a function of ξ in Figure 2c, are always lower than 2 and slightly decrease with ξ, indicating non-compact clusters that become more branched at increasing the lysozyme concentration and therefore the amount of protein adsorbed to AuNPs. In fact, when deposited on silicon substrates (SEM images of Figure 1d) AuNPs aggregates appear mainly disposed as single layers while superimposed AuNPs can be rarely found, as expected for loose and branched three-dimensional structures.

More insights on the short-range interparticle correlations are obtained by analyzing the structure factors S(q) of the samples with ξ≥1200, where the formation of fractal objects is clearly observed. To derive S(q), we divided the scattered intensity curves of samples by the form factor of single AuNPs, measured on the stock solution [30,42]. The resulting curves are reported in Figure 2d. The structure factors were fitted to a sticky hard spheres model [28] to extrapolate the effective radius *R* of the interacting colloids (Figure 2e). This parameter indicates the distance at which particles start to repel each other and it is assumed to describe the halved average center-to-center distance between nearest-neighbor AuNPs [30]. We then estimated the interparticle distance, i.e., the average distance between the gold surfaces of adjacent particles, by:

(2) $$d=2(R-R_{0}).$$

where $R_{0}$ is the radius of the bare AuNPs colloids, obtained by fitting the SAXS curves measured on the AuNPs stock solution to a spherical form factor [27] (Figure 2b). The obtained values of interparticle distance are reported as a function of ξ in Figure 2f. The distance between adjacent AuNPs is of few nanometers, consistent with the size of ∼3 nm of folded lysozyme proteins [19], and increases for the highest ξ values, when the largest amount of protein adsorbs to AuNPs and larger, more branched aggregates are formed.

Based on the analyses as a function of the protein concentration here reported, we identified the threshold number ratio ξ=1100, at which the aggregation of AuNPs is activated. At this number ratio, ∼50 protein molecules adsorb to each AuNPs [19] and the average distance between them can be estimated to ∼14 nm, by approximating the unoccupied regions of the AuNPs surface around each molecule with equivalent circles. For the following part of our investigation, aimed at studying the influence of pH and temperature on the clusters morphology and optical properties, we selected three number ratios: ξ=800, just before the threshold, ξ=1200, at the very beginning of the aggregation, when small AuNPs clusters are formed, and ξ=2500, corresponding to the formation of larger and branched aggregates that are stable in solution for the whole duration of the experiments.

### 3.2. Aggregates at Different pH

Here, we investigate the structure of the AuNPs clusters obtained by protein-mediated aggregation at varying the pH of the solution. To this aim we changed the pH both towards acid and basic pH values, by selecting for each range the number ratios at which we expect significant variations in the assemblies morphology.

**Figure 3:**
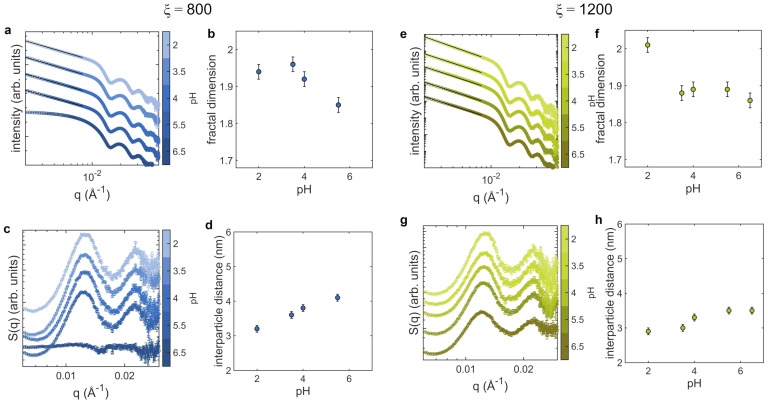
SAXS analysis of the AuNPs clusters morphology as a function of pH in acidic conditions (pH between 2 and 6.5), performed at 25 °C for two different lysozyme-AuNPs number ratios, ξ=800 and ξ=1200. (**a**,**e**) SAXS curves (circles) and power-law decay fitting curves (black lines); (**b**,**f**) fractal dimension as a function of pH; (**c**,**g**) structure factors; (**d**,**h**) interparticle distance as a function of pH. SAXS curves and structure factors are shifted vertically for clarity.

At first, we consider ξ=800 and ξ=1200, that at pH 6.5 are the number ratios right before and right after the aggregation threshold (Figure 1a), for which we analyzed the acidic range, down to pH 2.0. The corresponding scattering curves and structure factors, together with the plots of the fractal dimension and interparticle distance as a function of pH are reported in Figure 3.

In the case of ξ=800, at pH 6.5 the SAXS curve (Figure 3a) shows the classical plateau in the low-*q* region, indicating non-interacting AuNPs. Upon lowering the pH, the shape of the scattering curve in the low-*q* region changes, highlighting the formation of fractal aggregates. The extrapolated fractal dimensions, reported in Figure 3b, rise with pH up to the maximum value $D_{f}∼2$ at pH 2, suggesting the increasing density of the clusters. The structure factors, reported in Figure 3c, exhibit consistent variations with pH. At pH 6.5, S(q)∼1, while by lowering the pH the peak corresponding to short-range interparticle correlation appears and its maximum progressively shifts towards higher *q*. The significant decrease of the interparticle distance at lowering pH (Figure 3d) corroborates this observation.

In the case of ξ=1200, AuNPs are already organized in small clusters at pH 6.5, as reported in SEM images (Figure 1d). The scattering curves as a function of pH of Figure 3e show an increasing slope in the low-*q* region of the log-log plot, that becomes particularly pronounced at pH 2. From the trend of the fractal dimension (Figure 3f) it is evident indeed an abrupt increase of $D_{f}$ at the lowest pH. Concerning the structure factor (Figure 3g), also in this case we observe a shift of the peak towards high *q* values with decreasing pH and a decrease of the interparticle distance (Figure 3h).

**Figure 4:**
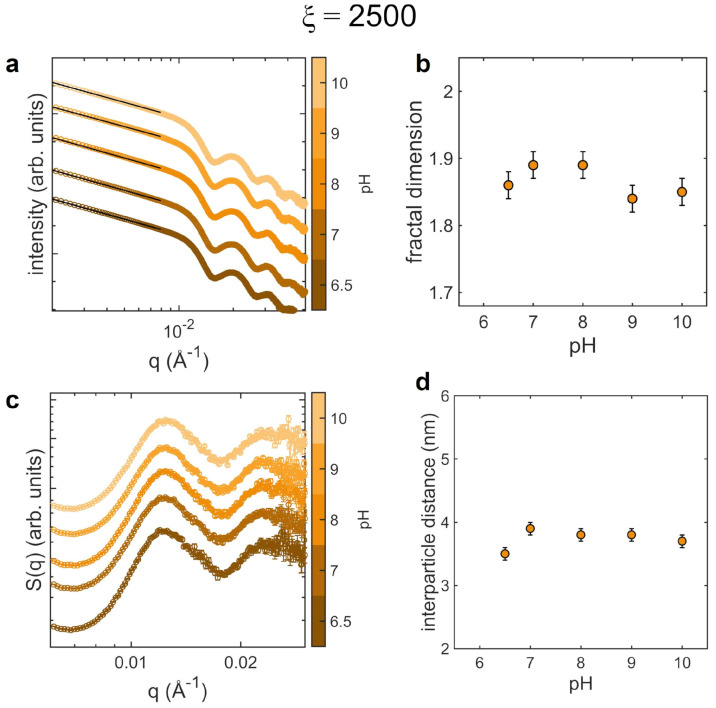
SAXS analysis of the AuNPs clusters morphology as a function of pH in basic conditions (pH between 6.5 and 10), performed at 25 °C for ξ=2500. (**a**) SAXS curves (circles) and power-law decay fitting curves (black lines); (**b**) fractal dimension as a function of pH; (**c**) structure factors; (**d**) interparticle distance as a function of pH. SAXS curves and structure factors are shifted vertically for clarity.

We also investigate the structure of AuNPs aggregates when pH is changed to basic values, ranging from 6.5 and 10. In this case we select ξ=2500, that corresponds at pH 6.5 to the formation of clusters with average hydrodynamic diameter of ∼ 270 nm. The SAXS data and analysis are reported in Figure 4. The scattering curves (Figure 4a) do not display evident variations of the slope in the low-*q* region. Consistently, the fractal dimension (Figure 4b) remains quite stable in the overall range of pH, with slightly lower values at pH 9 and 10. The analysis of the structure factors (Figure 4c,d) shows that the average interparticle distance increases with pH, unlike the case of acidic pH.

**Figure 5:**
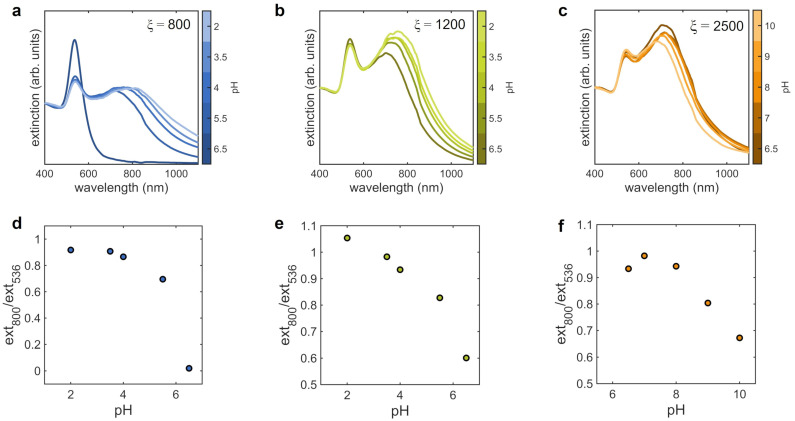
Optical properties of the AuNPs clusters at varying the pH of the solution for the three lysozyme-AuNPs number ratios analyzed, ξ=800, ξ=1200 and ξ=2500. (**a**–**c**) Extinction spectra at varying pH; (**d**–**f**) calibration curves as a function of pH, derived from the extinction spectra by the ratio between the extinction values at 800 and 536 nm. The temperature was kept at 25 °C during experiments.

The optical properties of the samples in the different pH analyzed are reported in Figure 5, together with the corresponding plots of the ratio between the extinction intensities at 800 and 536 mm as a function of pH. For ξ = 800 (Figure 5a), the shape of the extinction spectra change from the typical plasmonic profile of single AuNPs, at pH 6.5, to the broader two-bands shape of nanoparticle aggregates, at lower pH. The plasmonic band increasingly spreads towards higher wavelength by lowering pH suggesting the increased weight of coupled plasmons in acidic environment, similarly to what occurs at increasing number ratios (see the plasmonic profiles at ξ=5000 and ξ = 10,000 in Figure 1b). However, along with the increased size of clusters, the higher density of AuNPs (increased fractal dimension, Figure 3b) contributes in this case.

In the case of ξ=1200 (Figure 5b), the intensity of the peak of the extinction spectrum corresponding to the interparticle plasmonic modes, at ∼800 nm, increases with decreasing pH. This indicates the enhanced plasmon coupling induced by the closer proximity of AuNPs, consistently with the increasing of the fractal dimension (Figure 3f) and with the reduction of the interparticle distance (Figure 3h).

For ξ=2500 (Figure 5c), studied in basic pH conditions, the intensity of the extinction band of the interparticle modes decreases with increasing pH and its maximum shifts towards smaller wavelegths. This behavior is opposite to that observed for ξ=1200. At the same time, the intensity of the peak at ∼536 nm, corresponding to the LSPR of single AuNPs, increases. These spectral changes indicate the reduction of the plasmon coupling between AuNPs composing the assemblies and therefore suggest a possible detachment of some AuNPs from the clusters when pH is increased. The three calibration curves of Figure 5d–f reveal that it is possible to select different working regions, intended as the range of pH where the sensitivity of the extinction ratio is maximum, depending on the number ratio. In the case of ξ=2500, the calibration curve shows a non-monotonic trend in the range of pH between 6.5 and 8.0, outside the working range. This is due to the weak dependence of spectra on pH, making significant other fluctuations in the spectra that could affect the value of the extinction ratio. However, this aspect does not limit the feasibility of pH measurements, since the sample at ξ=2500 would be employed in the pH range between 8.0 and 10.0.

### 3.3. Aggregates at Different Temperatures

In this section, we analyse the effects of temperature on the structure and optical properties of the nanomaterial for the three selected protein-AuNP number ratios. Starting from 25 °C, we increase the temperature of the samples up to 80 °C. In the case of ξ=800, the temperature increase has no effect on the stability of the colloidal dispersion, as reported in Section S2 of the Supplementary Materials. SAXS data and extinction spectra demonstrate indeed that AuNPs do not aggregate for all the temperatures analyzed.

**Figure 6:**
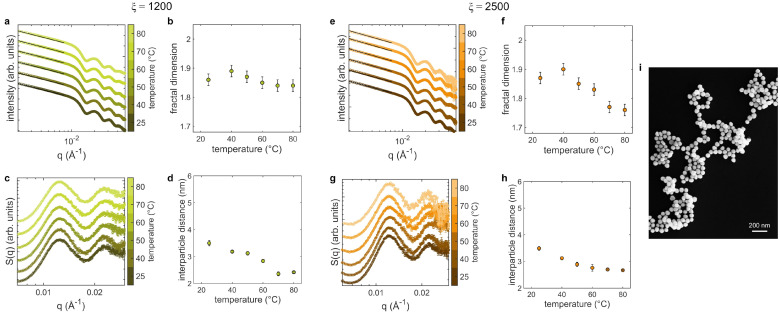
SAXS analysis of the AuNPs clusters morphology as a function of temperature (from 25 °C to 80 °C), performed at pH 6.5 for two different lysozyme-AuNPs number ratios, ξ=1200 and ξ=2500. (**a**,**e**) SAXS curves (circles) and power-law decay fitting curves (black lines); (**b**,**f**) fractal dimension as a function of temperature; (**c**,**g**) structure factors; (**d**,**h**) interparticle distance as a function of temperature; (**i**) representative SEM image of the assemblies obtained for ξ=2500 and deposited at 80 °C. SAXS curves and structure factors are vertically shifted for clarity.

In the case of aggregates already formed in the solution, for ξ=1200 and ξ=2500, the SAXS analyses at varying temperature are reported in Figure 6. The low-*q* range of the scattering curves of the sample with ξ=1200, containing the smallest assemblies, reveals a decrease of the fractal dimension with increasing temperature that is more pronounced starting from from 50 °C onward (Figure 6a,b). The corresponding structure factors (Figure 6c) show a progressive shift of the peak toward high *q* values, pointing to a decrease of the average distance between AuNPs (Figure 6d). A similar behaviour of the structural and morphological properties is observed in SAXS data for ξ=2500 (Figure 6e–h). Also in this case, the fractal dimension lowers with temperature down to the minimum value $D_{f}∼1.75$ and the interparticle distance displays a clear reduction at 60 °C. This marked reduction of the fractal dimension is confirmed by the branched and open structure of the assemblies at high temperature, as evidenced by the representative SEM image of Figure 6i, acquired on the sample deposited on a silicon substrate after increasing the temperature to 80 °C.

**Figure 7:**
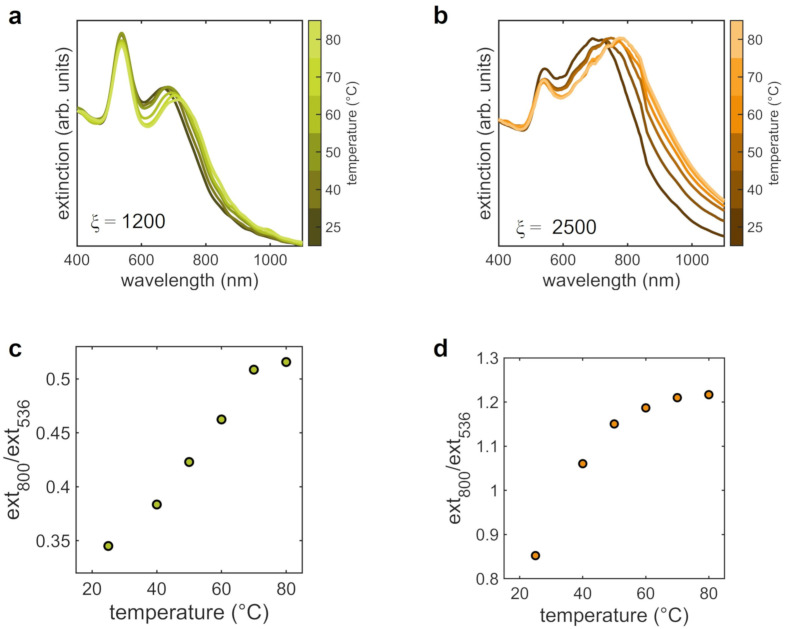
Optical properties of AuNPs clusters at varying the temperature of the solution for the two lysozyme-AuNPs number ratios analyzed, ξ=1200 and ξ=2500. (**a**,**b**) Extinction spectra at varying temperature; (**c**,**d**) calibration curves as a function of temperature, derived from the extinction spectra by the ratio between the extinction values at 800 and 536 nm. The pH of the solutions was 6.5 during experiments.

The analysis of the optical properties of samples with ξ=1200 and ξ=2500 as a function of temperature are reported in Figure 7 in terms of extinction spectra and corresponding plots of the ratio between the extinction values at 800 and 356 mm. In both cases, in the extinction spectra the LSPR of single AuNPS (peak at ∼536 nm) slightly decreases at increasing temperature. At the same time, the interparticle plasmonic band, at ∼800 nm, broadens towards higher wavelengths, more markedly for ξ=2500. The redshift and slight widening of this band at ξ=1200 suggests the enhanced coupling between AuNPs, as evidenced by the concomitant reduction of the interparticle distance (Figure 6d). For ξ=2500 instead, the more pronounced broadening might indicate an increased size of the AuNPs clusters promoted by the temperature.

## 4. Discussion

In this work, we performed a detailed structural investigation on a self-assembled plasmonic nanomaterial with the purpose of clarifying the effects of the assembly morphology on its optical properties. We considered three external stimuli—analyte concentration, pH and temperature—of pivotal interest for possible sensing applications. To this aim, we focused on the case-study of the electrostatic, protein-mediated assembly of AuNPs. The peculiar characteristic of our system is the formation of long-lived clusters with finite size. This can occur only in the presence of competing interaction forces that result in a limiting mechanism for the assembly process [43]. In our specific case, the aggregation is triggered by the adsorption of a positively charged protein, lysozyme, to the oppositely charged surface of anionic AuNPs [19]. This gives rise to the formation of colloidal particles with inhomogeneous surface charge, i.e., with surface patches that are oppositely charged with respect to the net charge of the particle. The interaction between such patchy colloidal particles may show a significant attractive component—arising from short-range, local interaction between oppositely charged patches on the approaching particles—even if the net charges on the two particles have the same sign. This results in a complex aggregation phenomenology that leads to the formation of clusters with finite-size, as pointed out by several experimental studies on the self-assembly of charged colloidal particles, when mixed with oppositely charged polymers, macromolecules or nanoparticles in an aqueous solvent [19,44,45,46]. The final size of the formed aggregates for given values of pH, temperature, ionic strength and concentration is controlled by the charge ratio between the adsorbing species and the colloids, and therefore by the amount of adsorbate present in the solution. By increasing the adsorbate content, the progressive reduction of the net charge of the primary colloids induces the formation of growing clusters. Close to the isoelectric point, where the charge of the adsorbed layer neutralises the original charge of the colloids, the aggregates reach their maximum size. For higher adsorbate content, the sign of net charge of the colloids can be reversed, leading to a reentrant condensation that results in the gradual decrease of the cluster size [47]. This phenomenology and the mechanisms that drive the self-limited aggregation can be described by the “charge-patch” interaction potential [47], originally proposed by Velegol and Thwar to describe the interaction between two inhomogeneously charged (patchy) colloidal particles in the Derjaguin approximation, i.e., when their size is larger than the electrostatic screening length of the colloidal suspension [48]. The two main parameters of the model are the average electrostatic surface potential ζ of two identical approaching particles with radius *a* and the corresponding standard deviation σ, that accounts for the charge inhomogeneity. The expression for the potential combines an attractive contribution, depending on σ, and a repulsive one, depending on ζ. Since the two components have different interaction ranges, they give rise to a potential barrier that two approaching particles must overcome to stick together. The height of the barrier is given, in units of the thermal energy $k_{B}T$, by:

(3) $$V_{max}=\pi \varepsilon a\left\{\sigma^{2}\ln\left[1-{\left(\frac{\zeta^{2}}{\zeta^{2}+\sigma^{2}}\right)}^{2}\right]+2\zeta^{2}\ln\left(1+\frac{\zeta^{2}}{\zeta^{2}+\sigma^{2}}\right)\right\},$$

where ε is the permittivity of the dispersing medium. Here the term proportional to $\sigma^{2}$ is negative and the one proportional to $\zeta^{2}$ is positive; therefore, aggregation is induced when the surface charge inhomogeneity increases or the total absolute charge on the colloids surface diminishes. Moreover, $V_{max}$ is proportional to the radius of the approaching particles, indicating that aggregation is more favored when the curvature of the approaching surfaces is higher.

This charge-patch interaction model adequately decribes the protein-mediated aggregation of AuNPs exploited in the present work [19]. In fact, in our case, the colloidal particle are represented by AuNPs, whose citrate capping provides a homogeneous negative surface charge. The charge patches are formed when lysozyme, that is positively charged in the overall experimental condition explored, adsorbs to AuNPs. Due to the small portion of the AuNPs surface area covered by each protein molecule (full coverage is obtained with ∼1800 molecules) we could finely tune the charge inhomogeneity by the lysozyme-AuNPs number ratio. Notably, this theoretical model introduces a limiting mechanism in the aggregation process even if accounting only for local interactions. In this respect, charge-patch interactions can not be framed within the two main mechanisms commonly identified to describe self limited aggregation, namely reaction limited aggregation (RLA) and diffusion limited aggregation (DLA) [49,50]. In fact, in RLA the final size of clusters is determined by the balance between attractive and repulsive interactions that depends on the size of the forming aggregates. This is excluded when the Derjaguin approximation holds. In the case of DLA, the limiting factor is the lowered probability for two particles to collide, which decreases when colloids aggregate. This mechanism does not account for reentrant condensation, that instead occurs in our case [19].

The formation of assemblies with increasing size as a function of protein concentration is evident from DLS and SAXS measurements of Figure 1 and Figure 2. Notably, at increasing the protein amount we observed an higher spacing between AuNPs in the cluster, from 3.5 up to 5.3 nm. The values of the interparticle distances are comparable with the size of lysozyme and consistent with a progressive accumulation of proteins on the colloids surface. The fractal dimension of the assemblies falls between 1.85 and 1.87, depending on the number ratio ξ. These values do not lie in the ranges associated to RLA ($1.9\leq D_{f}\leq 2.1$) and DLA ($1.7\leq D_{f}\leq 1.8$) models, witnessing the different mechanism that arrests the growth of clusters. The low density of such fractal aggregates can be explained if considering the dependence of the potential barrier between approaching particles on the surface curvature (Equation (3)). When aggregates are growing, the effective radius of curvature involved in the interactions is the local radius of curvature of the cluster. Hence, one approaching AuNPs sticks more likely to a sharp region of the cluster surface, where the barrier is lower, than to a flat one.

Proceeding on this line, we analyze the experiments on the cluster morphology at varying pH and temperature in the framework of the charge-patch interactions. For these investigations we selected three protein-AuNPs number ratios: 800, 1200 and 2500. The pH of the solution affects the protonation/deprotonation degree of the chemical groups that confer electrostatic charge to the proteins and to the AuNPs, namely amines and carboxyl groups. Therefore, this parameter directly affects the interaction strength between particles through their surface potential and surface charge inhomogeneity. The surface charge of AuNPs is owed to a capping of citrate molecules that include three carboxyl groups, whose $pK_{a}$ values occur at pH 3.1, 4.7, and 6.4, respectively [51]. This implies that the negative surface charge of AuNPs is gradually shielded with decreasing pH until it is completely neutralized at pH values lower than 3.1. Lysozyme, on the contrary, has one of the highest isoelectric points among proteins, occurring at pH 10.0 [52,53], due to the presence of a large number of positive amino acidic residues containing amino groups. Therefore its overall charge, that remains positive in the full range of pH employed in our experiments, is markedly positive in acidic environment and is almost neutralized in basic solutions [52,53]. On this basis, it is possible to frame the results obtained on the AuNPs clusters morphology at varying the pH of the solution in the charge-patch interaction model. In acidic environment, the surface charge inhomogeneity σ increases (higher charge of the positive patches) and the surface potential ζ of the primary colloids diminishes (lowering of the negative charge of the citrate capping). As a consequence, the lowering of the potential barrier (Equation (3)) enables the onset of the clustering in samples with small amount of protein (ξ=800, Figure 3). Coherently, the interparticle distance progressively lowers, down to 2.9 nm (ξ=1200, Figure 3). It is worth mentioning that lysozyme is a robust protein that retains its globular folding down to pH 3.0 [54]. Therefore, here we exclude a contribution of protein denaturation in the reduced spacing between AuNPs with decreasing pH, that is instead owed to the strengthening of the interaction between nearest-neighbor AuNPs. Moreover, at low pH values the fractal dimension of the clusters increases up to $D_{f}≃2.0$. The lowering of the potential barrier, increases the probability for AuNPs to stick also to regions of the forming clusters with higher radius of curvature, making them more compact. This is particularly evident at the lowest pH, for which the negative charges on the AuNPs surface are almost completely neutralized.

In basic solutions the behavior is opposite. The negative charge of AuNPs is preserved and actually increased, while the net charge of the protein is reduced, leading to a decrease of the inhomogeneity of the surface charge. Therefore, the attractive contribution to the pair interaction potential diminishes and the height of the potential barrier raises. This weakening of the interaction strength is reflected in the augmented interparticle distance due to the higher repulsion between colloids that could eventually lead to the detachment of some AuNPs from the clusters, but is not sufficient to induce the complete disaggregation. The corresponding fractal dimension diminishes significantly at pH 9 and 10, indicating loosen structures.

All the discussed structural modification of the assemblies induced by pH variations are reflected by their optical properties (Figure 5). The aggregation of AuNPs at acidic pH leads to a redshift and broadening of the band at ∼800 nm, more pronounced for ξ=800, indicating the presence of a higher number of coupled plasmons as a consequence of the formation of larger and denser aggregates promoted by the drop of the energy potential barrier. This implies the superposition of several interparticle plasmonic modes corresponding to different coupling strengths between plasmons [33]. In the case of ξ=1200, the increased intensity of the band at ∼800 nm, corresponding to coupled plasmons, is not accompanied by a marked widening beyond 900 nm. This implies that the size of the assemblies does not increase significantly, and the observed modification in the plasmon coupling is mainly due to the reduced interparticle distance and increased fractal dimension. On the contrary, in basic environment we observe a re-entrant behaviour of the plasmon coupling represented by the blueshift of the band at high wavelengths, due to the weakened interactions between colloids.

The role of temperature mainly consists in enhancing the diffusivity of the single AuNPs and of the clusters in the dispersion; therefore, the fraction of particles that have sufficient energy to overcome the potential barrier increases [55,56]. In fact, SAXS and SEM experiments performed on the assemblies point out that AuNPs remain aggregated (Figure 6) and reveal the presence of assemblies with an average size that is larger with respect to that of the original clusters formed at 25 °C. Moreover, by increasing temperature, the increase of the height of the potential barrier determines the opening and loosening of the aggregates structure, as witnessed by both SEM (Figure 6i) and by the marked reduction of the fractal dimension down to 1.76 in the case of ξ=2500. The observed decrease of the interparticle distance with increasing temperatures might appear to be contradictory in this framework. Nevertheless, an important aspect that must be taken into account is the effect of temperature on the protein structure. In fact, the protein unfolding, occurring starting above 50 °C [57], determines the reduction of the lysozyme thickness that results in a reduced interparticle spacing. Furthermore, the onset of other types of interactions between the colloids following protein denaturation—such as hydrophobic or covalent interactions as a result of the exposure of hydrophobic residues to the solvent or the rupture of disulfide bridges [15]—could contribute to the decreased interparticle distance. Even in this case the optical features of the assemblies strictly follow the colloidal aggregation dynamics. The enhancement of the plasmon coupling due to the lowered distance between the AuNPs and the increase of the average cluster dimension are highlighted by the redshift and broadening of the extinction band at 800 nm, particularly evident for ξ=2500 (Figure 7).

**Figure 8:**
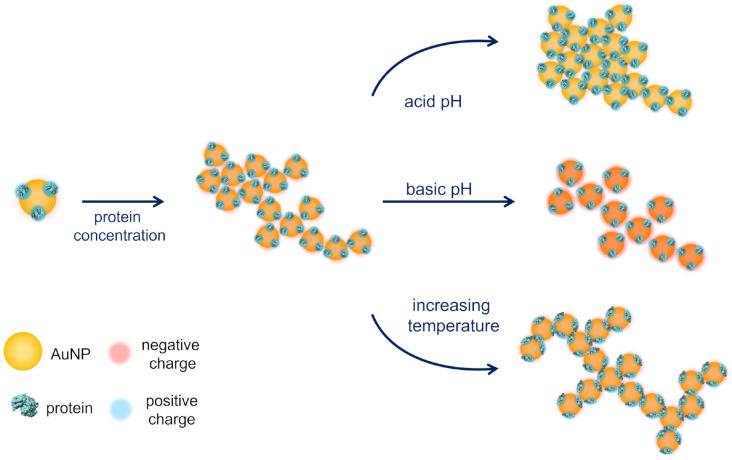
Schematic illustration of the observed structural variation of the protein-AuNPs assemblies in response to the different external stimuli analyzed: protein concentration, pH and temperature.

A scheme of the properties of the fractal assemblies as function of the different experimental parameters analyzed in this work is reported in Figure 8. In summary, at fixed temperature, if the attractive component of the potential (σ) is increased (acidic pH), aggregation is prompted, AuNPs can stick also to regions with high radius of curvature (resulting in increased fractal dimension), and the interparticle distance is lowered. Instead, at fixed interaction potential, when temperature is increased the aggregate results more banched due to the higher energy of interacting particles that stick immediately to the outer AuNPs of the clusters and do not fill the inner voids. These considerations about the influence of the external parameters on the fractal dimension of aggregates enable to interpret the near-field microscopy results previously reported on the aggregation of soft colloids mediated by charge-patch interactions [19,58,59,60].

The detailed analysis of the response to environmental stimuli proposed in the present work leads the way to develop plasmonic sensors based on the protein-mediated colloidal aggregation of AuNPs. To this aim, we derived from the extinction spectra different calibration curves for protein concentration (Figure 1c), pH (Figure 5d–f) and temperature (Figure 7c,d) by defining an experimental parameter, that is the ratio between the contribution of coupled plasmons at 800 nm and that of the single particle LSPR at 536 nm. In the case of protein concentration, we observe a strong sensitivity at extremely low amount of protein, between 17.2 and 25.8 nM. The potentiality of our approach becomes particularly evident when looking at the calibration curves as a function of pH and temperature. We can evaluate the sensitivity of the sensor from the steepness of the calibration curve as a function of the measured parameter. Given the different behavior of the assemblies depending on their initial morphology, it is possible to obtain various sensors with different dynamic range of sensitivity for the quantity of interest only by changing the number ratio ξ between protein and AuNPs. In the case of pH, we have maximum sensitivity in the pH range between 4 and 6.5 for ξ=800, between 2 and 6.5 for ξ=1200 and between 8 and 10 for ξ=2500. In the case of temperature, we found maximum sensitivities in the ranges 25 ÷ 60 °C and 25 ÷ 50 °C for ξ=1200 and ξ=2500, respectively. Notably, both pH and temperature ranges includes physiological conditions, important for sensing applications in the biomedical field. These findings highlight the flexibility of our system that allow to design sensing devices selecting “a priori” the working point more suited for specific applications.

## 5. Conclusions

The aggregation of plasmonic nanoparticles is a widely exploited phenomenon in the development of novel and highly performing sensing devices. In particular, the realization of colorimetric sensors relying on the controlled aggregation of plasmonic nanoparticles has been employed in the detection of various analytes such as viruses [61], hormones [62], food contaminants [63], opioids [64] as well as for the determination of variations in environmental parameters as temperature [65]. In this work, we focused on the effects on the optical properties of the plasmonic aggregates structure rearrangement upon variation of external parameters such as analyte concentration, pH and temperature. Compared to the other systems, the peculiarity of the proposed nanosensor lies in its high versatility. In fact, the same sensing platform can be exploited for measuring three different parameters. Moreover, by acting on the protein-AuNPs number ratio, and thus on the assembly of the original system, it is possible to move the working point and sensitivity of the sensor in different regions of interest. In addition to these assets, our approach can be transferred to other biologically relevant macromolecules with responsiveness in different ranges of pH and temperature, leading to a considerable widening of the possible applications. In more detail, we analyzed the morphology of clusters in terms of fractal dimension and interparticle distance and its dependence on the external stimuli by framing the experimental results in the charge-patch interaction theoretical model. We then interpreted the optical properties of the system on the basis of the studied morphological modifications. Experiments allowed us to build calibration curves as a function of the three parameters examined. The system demonstrated to be promising and highly flexible, allowing for selecting different working ranges, depending on the application of interest. Our results represent an important step towards the rational design of plasmonic sensors based on the self-assembly of nanoparticles.

## Data Availability

The data presented in this study are available from the corresponding authors upon reasonable request.

## Supplementary Materials

The following are available online at https://www.mdpi.com/article/10.3390/nano12091529/s1, Figure S1: Study of the time evolution and stability of the plasmonic aggregates, Figure S2: Analysis of the AuNPs clusters as a function of temperature for the number ratio ξ=800.
