# Supplementary material for: Responsivity of Fractal Nanoparticle Assemblies to Multiple Stimuli: Structural Insights on the Modulation of the Optical Properties
Source: Nanomaterials (Basel). 2022 May 1;12(9):1529. doi: 10.3390/nano12091529 (PMC9099587; doi:10.3390/nano12091529)
Supplement: Supplementary file 1 [file nanomaterials-12-01529-s001.zip › nanomaterials-1683249 - Supplementary Materials.pdf]

Supplementary Materials: Responsivity of fractal nanoparticle assemblies to multiple stimuli: structural insights on the modulation of the optical properties

Angela Capocefalo *, Thomas Bizien, Simona Sennato, Neda Ghofraniha, Federico Bordi and Francesco Brasili *

S1. Time evolution and stability of samples

In Figure S1 we report the analysis of the stability in time of the lysozyme-AuNP samples, evaluated by DLS and extinction spectroscopy. We selected three number ratios ξ (1100, 1200 and 2500) and we monitored the evolution in time for 100 min.

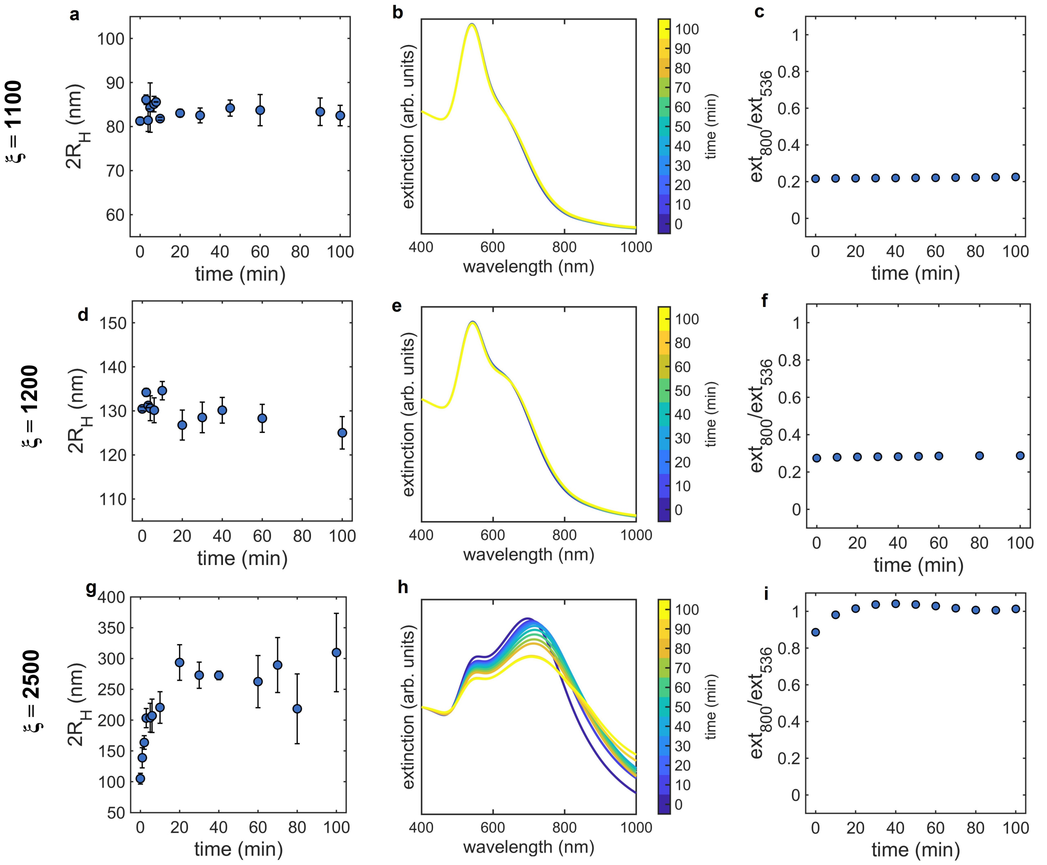

Figure S1. Study of the time evolution and stability of the plasmonic aggregates measured by DLS and extinction spectroscopy for three selected number ratios: $\xi = 1100$, $\xi = 1200$ and $\xi = 2500$. **(a,d,g)** Hydrodynamic diameter as a function of time; **(b,e,h)** extinction spectra at varying time; **(c,f,i)** ratio between the extinction values measured at 800 nm and 536 nm, as a function of time.

S2. SAXS curves and extinction spectra at varying temperature for the sample with ξ=800

The SAXS curves and the extinction spectra measured at varying temperature on the sample prepared with lysozyme-AuNPs number ratio $\xi = 800$ are reported in Figure S2.

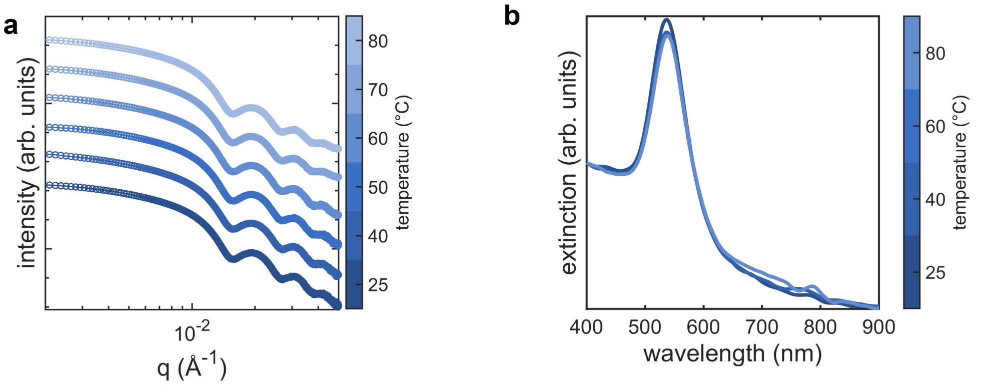

Figure S2. Analysis of the AuNPs clusters as a function of temperature (from 25 °C to 80 °C), performed at pH 6.5 for the lysozyme-AuNPs number ratio $\xi = 800$. **(a)** SAXS curves at varying temperature, the curves are vertically shifted for clarity; **(b)** extinction spectra at varying temperature, the spectra are normalized to the extinction at 400 nm.
